# Sleep pattern, healthy lifestyle and colorectal cancer incidence

**DOI:** 10.1038/s41598-022-21879-w

**Published:** 2022-10-31

**Authors:** Jie Chen, Nanqian Chen, Tao Huang, Ninghao Huang, Zhenhuang Zhuang, Hailun Liang

**Affiliations:** 1 School of Public Administration and Policy, Renmin University of China, 59 Zhongguancun, Beijing, 100872 China; 2 Department of Epidemiology and Biostatistics, School of Public Health, Peking University, Beijing, China; 3 Department of Global Health, School of Public Health, Peking University, Beijing, China; 4 Key Laboratory of Molecular Cardiovascular Sciences (Peking University), Ministry of Education, Beijing, China; 5 Center for Intelligent Public Health, Institute for Artificial Intelligence, Peking University, Beijing, China

**Keywords:** Cancer prevention, Colorectal cancer, Risk factors, Human behaviour

## Abstract

Researchers have identified an association between lifestyle factors and colorectal cancer (CRC) risk. This study examined the relationship between sleep patterns and CRC events. 392,252 individuals were sampled from the UK Biobank. Chronotype, sleep duration, insomnia, snoring, and excessive daytime sleepiness were combined to measure a healthy sleep score. A number of healthy sleep factors were defined, along with factors for healthy lifestyle scores. Using Cox proportional hazards regression, computed hazard ratios (HRs) were used to examine the associations between sleep patterns, healthy lifestyles, and the incidence of CRC. Healthy sleep scores were inversely associated with CRC events. The HRs for CRC were 0.90 (95% CI, 0.88–0.92) and 0.95 (95% CI, 0.92–0.98) for a 1-point healthy sleep score increase among males and females. When analyzing sleep components, sleeping 7–8 h/day, no frequent insomnia, no snoring, and no frequent daytime sleepiness were independently associated with a 9%, 14%, 8%, and 14% lower risk of CRC, respectively, whilst healthy lifestyle scores were inversely associated with CRC incidence across all models. Sleep pattern and lifestyle are significantly correlated with CRC risk. The healthier the subject’s lifestyle and sleep pattern, the lower their CRC risk.

## Introduction

Colorectal cancer (CRC) is the third most commonly occurring cancer among men and the second most commonly occurring cancer among women globally. Unsurprisingly, CRC produces a significant global burden. According to the WHO Cancer Research Center’s GLOBOCAN project, there are 1.8 million new CRC cases annually, with 880,000 CRC-related deaths in 2018 alone^1^. The probability of CRC survival highly depends on the stage at which it is diagnosed. At present, the 5-year survival rate of CRC in most European countries is between 60 and 69%^2^. Meanwhile, in the United States, although the 5-year survival rates of stage I colon cancer and rectal cancer > 90%, they drop to 11% and 15%, respectively, at stage IV^3^. Early screening and diagnosis are associated with a substantial decrease in CRC morbidity and mortality, thereby contributing to the optimal prognosis for survival and controlling treatment costs^4^. Moreover, several modifiable risk factors affect CRC in the long term; as such, when looking at preventing and reducing the general burden of CRC, such factors should be considered.

Aside from typically recommended lifestyle factors, increasing evidence has implicated that sleep pattern is related to adverse health events. Several lifestyle factors, including physical activity, diet patterns, smoking, drinking, and obesity, have been shown to influence CRC incidence^5–9^. In addition to these lifestyle behaviors, several studies have also put forward evidence that unhealthy sleep patterns, such as excessive daytime sleepiness and snoring, were risk factors for developing cancer^10–13^. In terms of CRC, a number of studies have identified an association between excessive sleep duration, insufficient sleep duration, and insomnia with CRC incidence^14–17^. Lin et al. investigated the relationship between CRC risk and sleep disorders, finding that the risk of CRC in patients with sleep disorders was significantly higher than in those without sleep disorders^18^. Sleep behaviors typically interplay with each other, whereas most previous studies focus on individual factor without considering the joint effect of other. Despite these advances, the following knowledge gaps remain to be filled. First, it is crucial to evaluate the combination of sleep behaviors on CRC incidence, since sleep-related factors are often interconnected and affect the development of a range of diseases. No study has taken several sleep pattern together and assessed their joint association with CRC risk. The interaction between sleep pattern and lifestyles, as well as the joint association of with CRC is still unclear. Second, studies with a large sample size and a prospective study design are often required to conclude sound evidence. Therefore, we aimed to estimate the association of the combination of the sleep pattern and overall lifestyle with the risk of CRC independently and jointly. Moreover, adding sleep pattern as a prevention target into traditional lifestyle scores and inclusion in behavioral recommendations could further reduce the risk of CRC associated with unhealthy lifestyle factors. With this in mind, we prospectively assessed the associations between a combination of major sleep behaviors and the incidence of CRC based on the UK Biobank dataset. We integrated data on several sleep behaviors and constructed a healthy sleep score framework to assess the relationship between overall sleep patterns and CRC risk. We are not aware of another similar study addressing such a novel scientific aspect of CRC risk prediction.

In addition, it is well-accepted that both predisposing and behavioral factors contribute to the development of CRC. Our study sample consisted of 392,252 individuals and we considered a comprehensive range of CRC risk factors, including traditional lifestyle factors, sleep patterns, family disease history, and other related covariates.

## Methods

### Study population

UK Biobank’s study design and methods have previously been described in detail^19^. UK Biobank is a large-scale biomedical database and research resource, containing in-depth genetic and health information. UK Biobank recruited > 500,000 community-based volunteers aged 40–69 years from across the UK between 2006 and 2010 (baseline visit). The study participants completed questionnaires and underwent physical measurements to gather information on their sleep patterns, lifestyles, and CRC outcomes. The UK Biobank research received approval from the North West Multicenter Research Ethical Committee. It should also be noted that all participants provided written informed consent to take part in the study.

In the current study, we excluded those samples with CRC at the baseline (N = 8669) and those with missing values for sleep (N = 90,105) and lifestyle variables (N = 11,478) at the baseline. This left a total of 392,252 participants for the main analysis. Among the participants, a total of 12,583 cases of CRC were recorded during a median follow-up of 8.51 years.

### Measurement

#### Assessment of sleep behaviors

The sleep behavior information was based on self-reported records collected from 2006 to 2010 during the baseline visit. Individual sleep behaviors include sleep duration, chronotype, insomnia, snoring, and daytime sleepiness^20,21^. *Sleep duration* was recorded as the number of sleep hours in response to the question ‘About how many hours sleep do you get in every 24 h? (include naps)’. *Chronotype preference* was measured by asking ‘Do you consider yourself to be (i) definitely a “morning” person, (ii) more a “morning” than “evening” person, (iii) more an “evening” than “morning” person, or (iv) definitely an “evening” person’. Information on *insomnia* was collected through the question **‘**Do you have trouble falling asleep at night or do you wake up in the middle of the night?’, to which subjects responded (i) never/rarely, (ii) sometimes, or (iii) usually. *Snoring symptoms* were obtained by asking **‘**Does your partner or a close relative or friend complain about your snoring?’ with responses of (i) yes or (ii) no. Finally, *daytime sleepiness* was coded based on the question ‘How likely are you to doze off or fall asleep during the daytime when you do not mean to? (e.g. when working, reading or driving)’, to which subjects responded (i) never/rarely, (ii) sometimes, (iii) often, or (iv) all of the time.

#### Definition of a healthy sleep score and sleep pattern

A healthy sleep score was created by combining five sleep components: sleep duration, chronotype preference, insomnia, snoring symptoms, and daytime sleepiness. The validity and utility was verified by Fan et al.’s^20^ study. Subsequently, the healthy sleep score has been deployed to analyze the association between sleep behaviors and the risk of incidence of type 2 diabetes^22^, heart failure^21^, arrhythmia^23^, hypertension^24^, cardiometabolic multimorbidity^25^, chronic kidney disease^26^, and mental disorders^27^ amongst others. These research findings are consistent with the definition and operationalization of the healthy sleep score framework, and the empirical results also confirm the reliability of the scoring framework. We then applied the same construction method to the prediction of CRC, as the first researchers to do so. The low-risk sleep factors in our study were defined as follows: sleep 7–8 h/day; early chronotype (“morning” or “morning than evening”); reported never or rarely insomnia symptoms; no self-reported snoring; and no excessive daytime sleepiness (“never/rarely” or “sometimes”). For each sleep behavior, low risk and high risk were coded as 1 and 0, respectively. The five scores were totalled to obtain a healthy sleep score ranging from 0 to 5, with higher scores indicating a healthier sleep pattern. After calculating the scores, the participants were divided into three groups: “Unfavorable sleep pattern (healthy sleep score of 0–1)”, “Medium sleep pattern (healthy sleep score of 2–3)” and “Favorable sleep pattern (healthy sleep score of 4–5)”.

#### Definition of a healthy lifestyle score

A range of lifestyle behaviors was selected based on measurement records or self-reported records at the baseline (2006–2010). In our study, a healthy lifestyle score was developed by combining physical activities, diet, drinking, smoking and obesity^28,29^: “ < 150 min/week moderate and < 75 min/week vigorous and < 150 min/week mixed (moderate + vigorous) physical activity”, “Diet ≥ 4 ideal food groups, ideal food groups were including fruits: ≥ 3 servings/day; vegetables: ≥ 3 servings/day; fish: ≥ 2 servings/week (counted by oily fish and non-oily fish); processed meat: ≤ 1 serving/week, unprocessed meat: ≤ 1.5 serving/week (counted by beef, lamb mutton, pork)”, “alcohol assumption < 14, 28 drinking-equivalents/day in women and men respectively ”, “Never or previous smoking”, “BMI (Body mass index) ≤ 25 kg/m^2^” were regarded as low-risk lifestyle factors. In the same way as noted above, low risk and high risk were coded as 1 or 0 and all the factors were totalled to generate a healthy lifestyle score ranging from 0 to 5, with a higher score indicating a healthier lifestyle. Again, the participants were divided into three groups: “Unfavorable lifestyle (healthy lifestyle score of 0–1)”, “Medium lifestyle (healthy lifestyle score of 2–3)” and “Favorable lifestyle pattern (healthy lifestyle score of 4–5)”.

#### Ascertainment of CRC events (outcomes)

The outcome of this study was the incidence of CRC (excluding baseline incidence). The outcome was defined according to the International Classification of Diseases, Tenth Revision (ICD 10). CRC events included malignant tumor of the colon (ICD code: C18.902) and malignant tumor of the rectum (ICD code: C20).

#### Covariant

The sociodemographic characteristics utilised in the study included age, sex, education, and household income. Additionally, family history (including parents and siblings) of CRC events was also controlled in the study. It should be noted that proven associations have been identified between these characteristics and the occurrence of colorectal cancer in previous studies^30,31^.

### Statistical analysis

The baseline characteristics of participants were described as means or percentages in each healthy sleep score category. Meanwhile, person-time (follow-up time) for each participant was calculated from the date of enrollment to the date of CRC diagnosis, death, or the end of follow-up, whichever occurred first. Cox proportional hazards models were used to calculate hazard ratios (HRs) featuring 95% confidence intervals for CRC events, with multivariable adjustments made for age, sex, education, household income, and family history of CRC.

To test the relationship between sleep and CRC incidence, we first estimated the HRs (95% CI) of CRC events per 1-number increment in healthy sleep score among different subgroups. We also analyzed the associations between each sleep pattern component and CRC events. Next, we estimated the impact of a 1-number change in healthy lifestyle score on the risk of CRC. Finally, we summed these two types of scores to explore the association between the summed score and CRC incidence. The assessment of benefit of adherence to a comprehensive healthy lifestyle and the incremental benefit after combining a healthy sleep pattern with a healthy lifestyle will shed light on the implication that a healthy lifestyle is the cornerstone of CRC prevention, whereas adherence to a healthy sleep pattern might complement the well-established lifestyle for the primary prevention. All of the reported P values were nominal and 2-sided, with P values of less than 0.05 considered statistically significant. All statistical analyses were performed using Stata software, version 15.0.

### Ethics approval

The UK Biobank study was approved by the National Information Governance Board for Health and Social Care in England and Wales, and the Community Health Index Advisory Group in Scotland and the North West Multicenter Research Ethics Committee. All participants gave written informed consent. This study was also approved by the Ethical Committee of Peking University (Beijing, China). In addition, all methods were carried out in accordance with relevant guidelines and regulations.

## Results

Table 1 details the baseline characteristics of the study according to the participants’ healthy sleep scores. Of the 392,252 study participants, 12,583 (3.21%) developed CRC during a median follow-up of 8.51 years. Meanwhile, 38.33% and 30.41% had healthy sleep scores of 3 and 4, respectively. Participants with higher healthy sleep scores also had higher healthy lifestyle scores.

**Table 1:** Baseline characteristics of participants according to the healthy sleep score.

Baseline characteristic		Healthy Sleep Score				
		0–1	2	3	4	5
Number of participants		17,164	77,231	150,366	119,275	28,216
Having low-risk sleep factors (%)	Sleep 7–8 h/day	4.50%	36.58%	66.46%	93.56%	100%
	Early chronotype	7.06%	31.02%	59.67%	86.29%	100%
	No frequently insomnia	1.12%	5.51%	14.65%	34.97%	100%
	No self-report snoring	5.02%	32.00%	60.68%	85.45%	100%
	No frequently daytime sleepiness	77.09%	94.89%	98.53%	99.72%	100%
Healthy lifestyle score		2.12	2.31	2.50	2.66	2.78
Age, year (%)	≤ 55	43.56%	42.13%	41.35%	43.26%	50.70%
	55–65	39.88%	39.84%	39.70%	38.04%	33.30%
	≥ 65	16.56%	18.02%	18.95%	18.69%	16.00%
Gender (%)	Female	47.06%	50.70%	55.69%	58.69%	54.79%
	Male	52.94%	49.30%	44.31%	41.31%	45.21%
Education (%)	Less than high School	68.13%	65.24%	62.17%	58.49%	54.78%
	High school or more	31.87%	34.76%	37.83%	41.51%	45.22%
Household income (%)	Less than 18,000	27.75%	22.89%	20.85%	18.45%	15.83%
	18,000–30,999	25.54%	25.88%	25.45%	24.89%	22.57%
	31,000–51,999	25.17%	26.35%	26.84%	27.12%	27.61%
	52,000–100,000	17.67%	20.04%	21.23%	23.06%	25.61%
	Greater than 100,000	3.87%	4.84%	5.63%	6.49%	8.39%
Family history of CRC (%)	No	93.27%	93.12%	93.18%	93.22%	93.52%
	Yes	6.73%	6.88%	6.82%	6.78%	6.48%

The results are presented as means (continuous variable) and percentage (category variable) according to healthy sleep score.

### Sleep pattern and risk of CRC events

As shown in Table 2, compared to participants with unfavorable sleep patterns, the CRC events hazard ratios of people with “medium” or “favorable” sleep patterns were 0.92 (95% CI, 0.85–1.00, P < 0.05), and 0.81 (95% CI, 0.74–0.87, P < 0.001), respectively. The results confirmed healthier sleep patterns led to a lower risk of CRC events. Consistent results were observed in strata of age, gender, education, household income, and family history of CRC (Table 3). Notably, we observed an interaction of each 1-number increment in healthy sleep score with all covariant variables. The HR for CRC events per 1-number increment in healthy sleep score was 0.95 (95% CI, 0.92–0.98, P < 0.005) among female participants and 0.90 (95% CI, 0.88–0.92, P < 0.001) among male participants. We found increment of healthy lifestyle score also led to a lower likelihood of developing CRC events across different age and education groups. With regard to household income, the HRs CRC events per 1-number increment in healthy sleep score for were 0.91 (95% CI, 0.88–0.95, P < 0.001), 0.94 (95% CI, 0.91–0.98, P < 0.005), 0.89 (95% CI, 0.85–0.92, P < 0.001), 0.91 (95% CI, 0.87–0.96, P < 0.001) among the four income groups, respectively. However, there were no significant interactions when participants’ household income was greater than 100,000 (P = 0.483). Besides, a 1-number increment in healthy sleep score reduced the risk of CRC events across participants’ different lifestyle scores. Last but not least, the HR for CRC events per 1-number increment in healthy sleep score was 0.91 (95% CI, 0.90–0.93, P < 0.001) among participants without a family history of CRC and 0.94 (95% CI, 0.89–1.00, P < 0.001) among participants with a family history of CRC.

**Table 2:** Hazard ratio (95% CI) of CRC events according to Sleep Score compared with participants with less than and equal to 1 combined sleep score.

Healthy sleep score		Hazard ratio^a^ (95% CI)	P value
Category	Score scope		
Unfavorable	0–1	1.00 (Ref)	
Medium	2–3	0.92 (0.85–1.00)	P < 0.05
Favorable	4–5	0.81 (0.74–0.87)	P < 0.001

392,252 participants included in the model.

^a^Hazard ratios (95% CI) were adjusted for age, sex, education, family history of CRC, healthy life style score.

**Table 3:** Hazard ratio (95% CI) of CRC events per 1-number increment in healthy sleep score among participants.

Subgroup	Hazard ratio^a^ (95% CI)	P value
**Gender**		
Female	0.95 (0.92–0.98)	P < 0.005
Male	0.90 (0.88–0.92)	P < 0.001
**Age, year**		
≤ 55	0.89 (0.86–0.93)	P < 0.001
55–65	0.92 (0.89–0.95)	P < 0.001
≥ 65	0.94 (0.91–0.98)	P < 0.005
**Education**		
Less than high school	0.91 (0.89–0.94)	P < 0.001
High school or more	0.92 (0.89–0.96)	P < 0.001
**Household income**		
Less than 18,000	0.91 (0.88–0.95)	P < 0.001
18,000–30,999	0.94 (0.91–0.98)	P < 0.005
31,000–51,999	0.89 (0.85–0.92)	P < 0.001
52,000–100,000	0.91 (0.87–0.96)	P < 0.001
Greater than 100,000	0.96 (0.87–1.07)	P = 0.483
**Family history of CRC**		
No	0.91 (0.90–0.93)	P < 0.001
Yes	0.94 (0.89–1.00)	P < 0.001
**Healthy lifestyle score**		
Unfavorable	0.92 (0.88–0.92)	P < 0.001
Medium	0.92 (0.89–0.94)	P < 0.001
Favorable	0.93 (0.88–0.99)	P < 0.05

^a^Hazard ratios (95% CI) were adjusted for age, sex, education, household income, family history of CRC, healthy lifestyle score.

**Figure 1:**
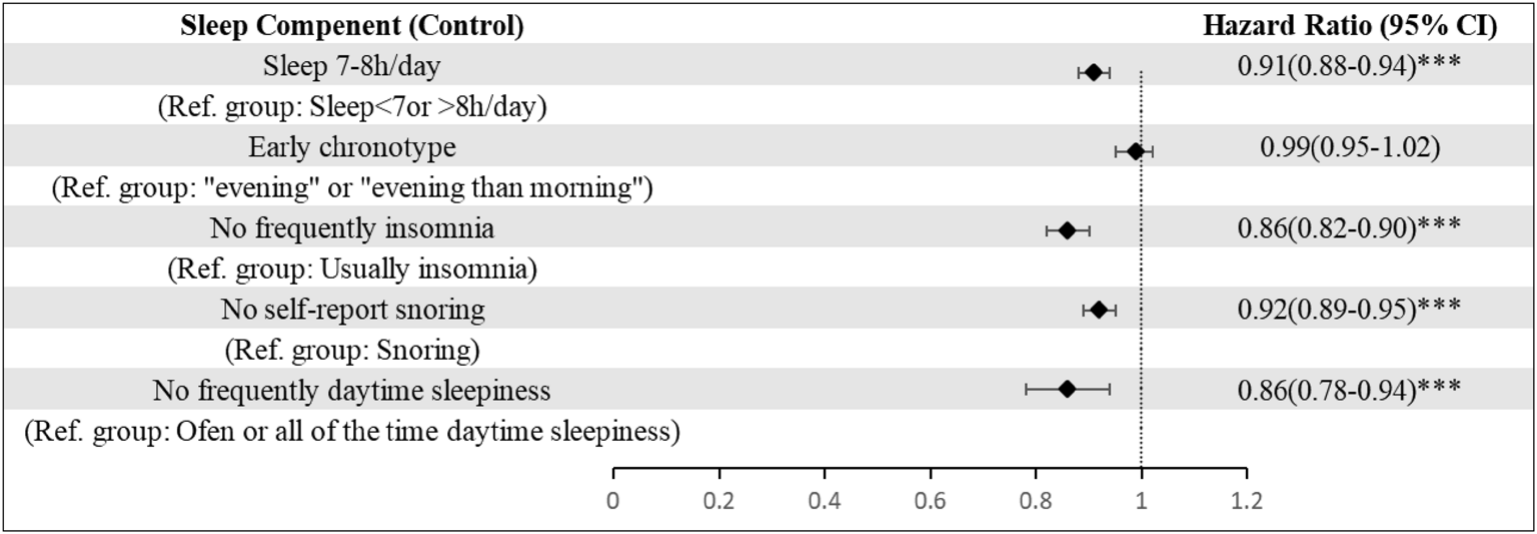
Hazard ratios (95% CI) of CRC events according to individual sleep components. 392,252 participants included in the model; hazard ratios (95% CI) were adjusted for age, sex, education, household income, family history of CRC, healthy lifestyle score.*, **, *** mean P-value < 0.05, 0.01, 0.001 respectively (the same below).

Figure 1 showed the associations between each individual sleep component and the risk of CRC events. The HR for CRC events was 0.91 (95% CI, 0.88–0.94, P < 0.001) among individuals who responded “sleep 7–8 h/day”, 0.86 (95% CI, 0.82–0.90, P < 0.001) among individuals who responded “No frequently insomnia” group, 0.92 (95% CI, 0.89–0.95, P < 0.001) among individuals who responded “No self-report snoring” group, and 0.86 (95% CI, 0.78–0.94, P < 0.001) among individuals who responded “No frequently daytime sleepiness” group. This meant that sleeping 7–8 h/day, no frequent insomnia, no self-reported snoring, and no frequent daytime sleepiness were each independently associated with a 9%, 14%, 8%, and 14% lower risk of CRC, respectively. Our study did not identify any significant relevance between participants’ sleep chronotype preference and risk of CRC.

### Lifestyle and risk of CRC events

Table 4 lists the HRs of CRC events per 1-number increment in healthy lifestyle score among participants. The HRs of CRC events per 1-number increment in healthy lifestyle score were 0.88 (95% CI, 0.85–0.90, P < 0.001) among female participants and 0.83 (95% CI, 0.81–0.85, P < 0.001) among male participants. When there was 1-number increment in healthy lifestyle scores, HRs were 0.87 (95% CI, 0.84–0.91, P < 0.001), 0.84 (95% CI, 0.81–0.86, P < 0.001), 0.86 (95% CI, 0.83–0.89, P < 0.001) among participants in year groups 1 to 3, respectively. We found that the HR for CRC events was 0.84 (95% CI, 0.82–0.86, P < 0.001) among participants whose education level was less than high school and 0.86 (95% CI, 0.83–0.89, P < 0.001) among participants whose education level was high school or above. In different income subgroups, a healthy lifestyle had the most significant effect on study subjects whose household income was greater than 100,000. A 1-number increment was associated with a 19% lower risk of CRC events among them. In addition, when healthy lifestyle had 1-number increment, the HR for CRC events was 0.85 (95% CI, 0.78–0.93, P < 0.001) among those participants without a family history of CRC, whilst it was 0.85 (95% CI, 0.80–0.90, P < 0.001) among participants with a family history of CRC. 1-number increment in healthy lifestyle score also reduced the risk of CRC events in participants with different sleep patterns.

**Table 4:** Hazard ratio (95% CI) of CRC events per 1-number increment in Healthy Lifestyle Score among participants.

Subgroup	Hazard ratio^a^ (95% CI)	P value
**Gender**		
Female	0.88 (0.85–0.90)	P < 0.001
Male	0.83 (0.81–0.85)	P < 0.001
**Age, year**		
≤ 55	0.87 (0.84–0.91)	P < 0.001
55–65	0.84 (0.81–0.86)	P < 0.001
≥ 65	0.86 (0.83–0.89)	P < 0.001
**Education**		
Less than high School	0.84 (0.82–0.86)	P < 0.001
High school or more	0.86 (0.83–0.89)	P < 0.001
**Household income**		
Less than 18,000	0.86 (0.83–0.89)	P < 0.001
18,000–30,999	0.84 (0.81–0.87)	P < 0.001
31,000–51,999	0.87 (0.84–0.91)	P < 0.001
52,000–100,000	0.83 (0.79–0.88)	P < 0.001
Greater than 100,000	0.81 (0.73–0.89)	P < 0.001
**Family history of CRC**		
No	0.85 (0.83–0.87)	P < 0.001
Yes	0.85 (0.80–0.90)	P < 0.001
**Healthy sleep score**		
Unfavorable	0.85 (0.78–0.93)	P < 0.001
Medium	0.85 (0.82–0.97)	P < 0.001
Favorable	0.86 (0.83–0.89)	P < 0.001

^a^Hazard ratios (95% CI) were adjusted for age, sex, education, household income, family history of CRC, healthy sleep score.

### The combined scores and risk of CRC events

**Figure 2:**
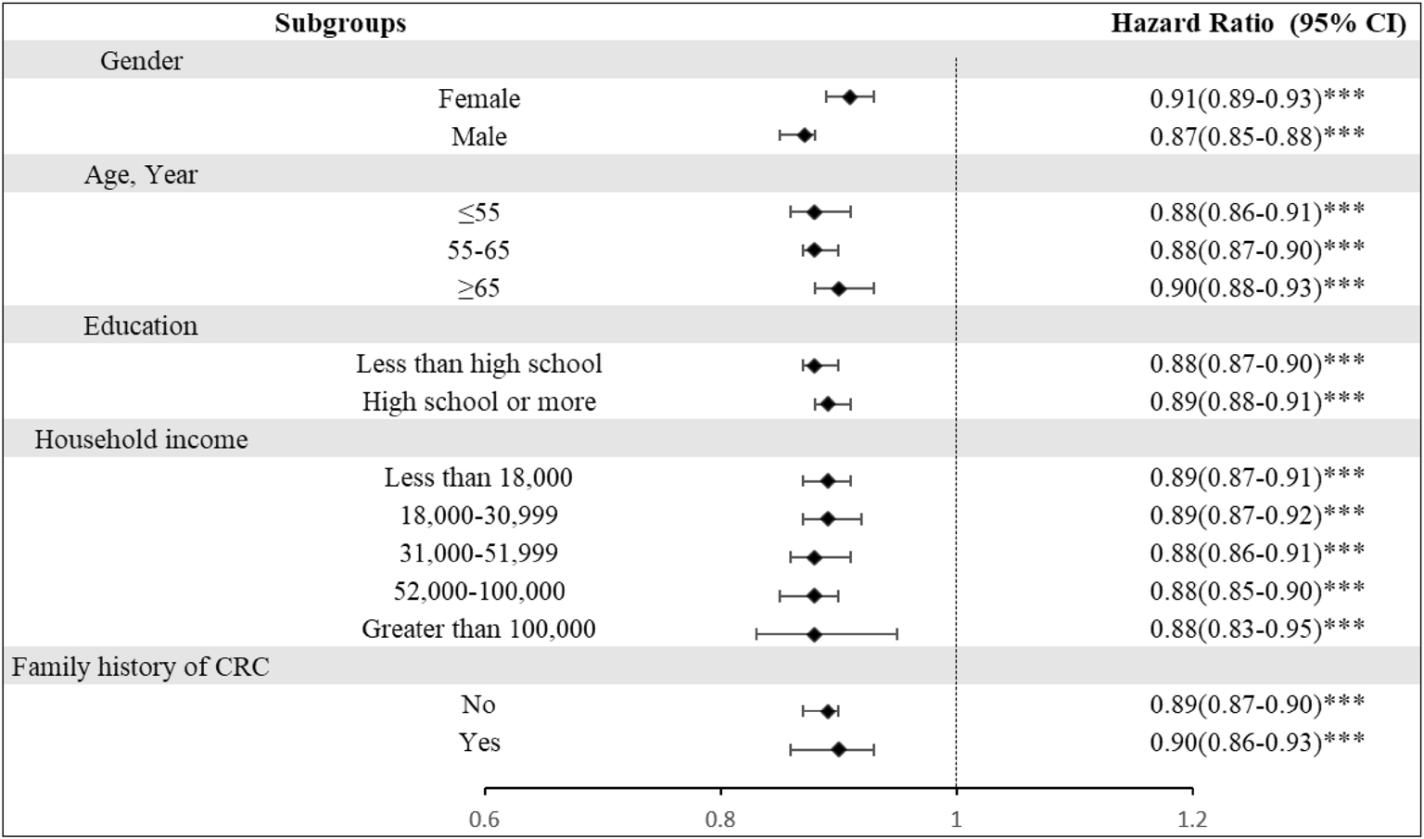
Hazard ratio (95% CI) of CRC events per 1-number increment in combined score among participants. Combined score: the sum of lifestyle score and sleep score; hazard ratios (95% CI) were adjusted for age, sex, education, household income, family history of CRC.

In addition to assessing the associations between sleep and lifestyle with CRC risk respectively, we further investigated whether there were any associations between combined scores (summed sleep scores and healthy lifestyle scores) and HRs for CRC events. As can be seen from the results in Fig. 2, a 1-number increment in combined score was associated with a lower risk of CRC in different subgroups (P < 0.001). Specifically, the HRs of CRC events per 1- number increment in combined score among the female and male groups were 0.91 (95% CI, 0.89–0.93, P < 0.001) and 0.87 (95% CI, 0.85–0.88, P < 0.001), respectively. Compared to those participants who did not have a family history of disease, the HR for CRC events of participants with a family history of disease was higher, at 0.90 (95% CI, 0.86–0.93, P < 0.001).

## Discussion

Sleep quality has been marked as a potentially modifiable risk factor for chronic disease incidence and mortality^16,32,33^. To the best of our knowledge, the present study is the first to investigate the association between a comprehensive sleep pattern made up of five components and the risk of CRC events. Moreover, the generalizability of the research findings was enhanced by the study’s large sample size and the representativeness of the UK Biobank sample. In this prospective cohort study of 392,252 adults, we found significant associations between sleep patterns and CRC risk. Regarding the healthy sleep score components, our study confirmed that sleep duration, insomnia, daytime dozing, and snoring can significantly influence the incidence of CRC. However, no association was found between chronotype preference and the HRs of CRC events. Similarly, the higher the healthy lifestyle score was, the lower the participants’ risk of CRC events. Besides, our data analysis also confirmed the combined scores of healthy sleep pattern and lifestyle contribute to reducing CRC risk.

By conducting several meta-analyses, previous studies concluded that higher sleep scores are associated with high blood pressure and cardiovascular disease^34–36^. However, a very limited number of studies have examined sleep disorders in cancer patients^37,38^, and there has rarely been a focus on the assessment of the general population^39^. Moreover, previous studies mainly evaluated the associations between individual sleep factors (i.e., duration, disruption) and CRC risk. No previous study has, to our knowledge, evaluated the whole sleep pattern as a CRC risk factor. The present study demonstrated that participants with higher healthy sleep scores were associated with a lower risk of CRC. Moreover, sleep pattern is known for its role in the regulation of hormones and metabolism, as well as in maintaining the immune system^40,41^. Specifically, the role of circadian rhythm and melatonin pathway disorders may explain the potential link between sleep and CRC risk. Unhealthy sleep patterns and disruptions to the circadian rhythm are related to both cancer processes^42^, due to an increase in oxidative stress and the response of inflammatory mechanisms, which can in turn cause a cascade in the cellular degeneration process^43^. Additionally, sleep deprivation may trigger high levels of tau protein, the growth of which leads to neurodegeneration^44^. A bidirectional association between neurodegeneration and cancer has recently been identified and related to the mechanisms of morphological alterations of mitochondria, telomere function, and genomic instability^45^.

The associations assessed between individual sleep factors and CRC in the present study were in line with the results of the previous research. Specifically, prior studies linked both short sleep duration and prolonged sleep with an elevated risk of CRC, thus providing support for the contention that sleep plays a role in colorectal carcinogenesis^46–48^. Based on a literature review of twelve studies, Mirghani et al. identified associations between short sleep duration (< 7 h) or prolonged sleep (> 9 h) and CRC^46^. Our research produced a similar result, in that individuals sleeping 7–8 h per day had a 9% lower risk of CRC, compared to people sleeping for a longer or shorter time. There are two plausible mechanisms underpinning this result: firstly, an unfavorable sleep duration may have enhanced cortisol secretion, increased insulin resistance^49^, and further led to weight gain^50,51^, obesity^50^, and diabetes^52^, which are all independent CRC risk factors^53,54^. Secondly, excessive sleep may lead to increased release of pro-inflammatory cytokines, especially IL-1 and THF, which play an important role in the growth of new tumors^14–16,55^.

An association between insomnia, the most common sleep disorder, and a risk of CRC has been identified in previous studies^15,17^. We derived similar results, in that reporting, no frequent insomnia was associated with a 14% lower risk of CRC, which was the most readily apparent effect on CRC risk of the five sleep pattern components. One potential explanation is that insomnia is very often associated with mood disorders, pain, and fatigue, which in turn trigger a multifactorial pathogenetic mechanism^56^. The series of mechanisms involves hormonal systems, different neurotransmitters, immune functions, excessive cytokine responses, interleukins, and tumor necrosis factor alpha (TNF-a). These alter the normal functioning of the hypothalamic–pituitary–adrenal (HPA) axis, which determines the physiological changes relevant to cancer that take place^57^.

With regard to the factor of snoring, our study findings support the significant association between snoring and CRC risk. Snoring is the key symptom of sleep apnea, and is typically used as a surrogate for sleep-disordered breathing, a common disorder associated with recurrent episodes of sleep disruption and intermittent hypoxemia^14^. Intermittent hypoxemia may lead to increase vascular endothelial growth and oxidative stress, which can damage DNA, RNA and lipids, which in turn can give rise to gene mutations and mitochondrial instability^58^. Moreover, in animal models, intermittent hypoxemia has been shown to promote tumor growth, possibly through the release of proangiogenic mediators that cause cell proliferation^59–61^.

Regarding daytime sleepiness, only two previous studies examined the association between the indicator and CRC risk^16,62^. We found similar results to these studies, in that excess daytime sleeping had a negative effect on the incidence of CRC. In a previous study, higher frequencies of sleepiness during the day and longer napping durations were associated with increased odds of developing CRC^16^. Like prolonged sleep, daytime napping has also been recognized as an indicator of health problems, with a notable relation to poor nighttime sleep, fatigue, and depression^16,63^. It appears that the interactions of and disturbances between the hypothalamic–pituitary–adrenal (HPA) axis and inflammatory cytokines determine whether an individual experiences daytime sleepiness. It should be noted that this creates systemic conditions that favor tumor growth^62,64^. However, further studies are needed to assess the physiological consequences of daytime napping on CRC to gain insights into the potential underlying biological mechanisms^16,63^.

In our subgroup analysis, we found healthy sleep scores had a greater impact on men than women. The HRs of developing CRC among women and men were 0.95 and 0.90, respectively, with 1-number increment in healthy sleep score. One potential explanation for this is that sleep induces a 24-h oscillation between predominant type 1 (proinflammatory) and type 2 (anti-inflammatory) cytokines, the presence of which increases the efficacy of immune responses^65^. The balance between type 1 and type 2 cytokines is gender dependent, with men exhibiting a greater type 2 response, which may also contribute to the differences observed between the two genders^65,66^. The results showed that as age increases, the positive effect of healthy sleep patterns on the risk of CRC decreases, which is consistent with the findings of the previous study^62^.

In addition to the findings related to the influence of sleep patterns on CRC, we also assessed the association between lifestyle (including physical activity, diet, smoking, drinking, and obesity) and the incidence of CRC. Many existing studies have concluded that lifestyle significantly influences CRC^67–69^. Similarly, our study found that a healthier lifestyle led to reductions in CRC incidence risk across all subgroups. 1- number increment of healthy lifestyle score was associated with a 12% and 17% lower risk of CRC events among women and men, respectively. The interplay between genetics, inflammatory and metabolomic responses, microbiota, immunity, behaviors, and environmental factors have important implications for how lifestyle impacts CRC incidence. Although the biological mechanism underlying the associations between physical activity and cancer risk remains unclear^69^, possible mechanisms include the beneficial influence of physical activity in circulating concentrations of insulin, stimulating insulin-related pathways, and reducing inflammation^70^. In terms of biological mechanisms underpinning the effects of diet patterns on CRC, evidence suggests that diet affects intestinal Fusobacterium nucleatum, which looks to play a role in CRC through the suppression of the host’s immune response to tumors^6^^7^. Moreover, obesity is a significant risk factor of CRC, which is associated with mitochondrial dysfunction, adipocyte hypertrophy, and oxidative and endoplasmic reticulum stress^69^. These responses promote increased adipokine secretion, proinflammatory signaling, and cell death, which in turn create a state of chronic low-grade inflammation^69^.

Several potential limitations to the present study merit discussion. It was difficult for us to measure the participants’ sleep duration, snoring, daytime sleepiness, and insomnia in an objective manner. Data based on self-reporting may result in some misclassification, which could bias the results in either direction. Additionally, unmeasured residual confounders cannot be ruled out as a possible explanation for the observed associations. Moreover, this study assessed the relationship between the baseline exposures and the outcome. Due to unavailability of data, it did not consider changes in exposure measurements during the follow-up. Besides, our study population was exclusively based in the UK, meaning that the results might not be generalizable to other countries.

The main strengths of our study are as follows: firstly, the implementation of a prospective design and long follow-up time minimized any potential selection or recall bias. Secondly, our study complemented the existing findings on individual sleep behaviors by jointly evaluating multiple sleep behaviors. Most of the previous studies only explored the relationship between sleep duration and CRC events; going beyond this, we added chronotype preference, snoring, insomnia, and daytime dozing as aspects of sleep patterns. Thirdly, when we analyzed the effects of sleep patterns on CRC risk and combined individual lifestyle with multiple components. Lastly, we used UK Biobank data; the high quality and large sample size of the data guaranteed the scientificalness of study results.

In summary, sleep pattern and lifestyle were significantly correlated with the risk of developing CRC. Accordingly, the healthier an individual’s lifestyle and sleep pattern are, the lower their risk of colorectal cancer. Our study results highlighted important implications that could serve as a resource for individuals and policy makers alike to improve CRC prevention efforts. We recommend that individuals keep a regular timetable of when they wake and go to sleep to guarantee they are sleeping for an appropriate length of time. It is also important to implement positive measures against sleep disturbance including snoring, insomnia and excessive daytime dozing. Meanwhile, developing a healthier lifestyle is vital for individuals to reduce their risk of CRC.

The findings also suggest that individuals should pursue healthy lifestyle practices throughout their lifetime. For governments, we recommend establishing a sound health information system taking knowledge dissemination as the core aim to allow more people to better understand how sleep patterns, lifestyle choices, and diseases are interrelated. We believe public health awareness can be effectively improved by governments if they enhance their efforts relating to health education. In the future, more research is warranted to evaluate whether sleep patterns are a novel risk factor for colorectal cancer. Finally, it is also necessary to explore the mechanisms underpinning how sleep patterns influence the risk of CRC events, which will support efforts to reduce the disease burden.

## Data Availability

Data are available in a public, open access repository. This research has been conducted using the UK Biobank Resource under Application Number 44430. The UK Biobank data are available on application to the UK Biobank (https://www.ukbiobank.ac.uk/).
